# Dissecting the dynamics of cell death pathways in Hirschsprung’s disease: a comparative analysis of viable and non-viable cells under proinflammatory conditions

**DOI:** 10.1007/s00383-024-05862-2

**Published:** 2024-11-03

**Authors:** Zhongwen Li, Johanna Hagens, Clara Philippi, Hans Christian Schmidt, Lucie Rohwäder, Pauline Schuppert, Laia Pagerols Raluy, Magdalena Trochimiuk, Konrad Reinshagen, Christian Tomuschat

**Affiliations:** Department of Pediatric Surgery, University Medical Center Hamburg-Eppendorf, Hamburg, Germany

**Keywords:** Organoids, RIK1, RIP3–caspase-3 assay, Hirschsprung’s disease, Apoptosis, Necroptosis

## Abstract

**Purpose:**

The present study explores the dynamics of cell death in Hirschsprung’s disease (HSCR) and control (CO) groups under inflammatory stress conditions.

**Methods:**

Using flow cytometry, we analyzed intestinal colonic organoid cultures derived from the ganglionic segment of the HSCR and CO groups. Our analysis focused on the quantification of RIPK1-independent and RIPK1-dependent apoptosis, as well as necroptosis in both viable and non-viable cells under acute and chronic inflammatory stress.

**Results:**

Our findings indicate that HSCR cells are particularly vulnerable to inflammation during acute proinflammatory stress, as evidenced by an increase in dead cells (Zombie +). Under chronic conditions, adaptive changes are observed in both HSCR and CO groups, indicating survival mechanisms. These adaptations are uniquely altered in HSCR, suggesting an impaired response to chronic inflammation. HSCR cells show significantly decreased RIPK1-dependent apoptosis in acute scenarios compared to chronic ones, unlike the CO group, implying varied responses to different inflammatory stresses. In non-viable cells, considerable changes in RIPK1-dependent apoptosis under chronic conditions in HSCR indicate a heightened inflammatory response compared to CO.

**Conclusion:**

This research provides insights into cell death regulation in HSCR under inflammatory stress by using patient-derived organoids, underscoring the complexity of its inflammatory response.

## Introduction

Hirschsprung disease (HSCR) is a congenital disorder characterized by a lack of ganglion cells in the enteric nervous system, affecting approximately one in 5,000 live births [1]. This condition leads to considerable gastrointestinal problems due to neuroblasts failing to migrate during embryonic development, resulting in various clinical symptoms based on the extent of intestinal involvement [2]. The primary treatment involves surgically removing the aganglionic section. Nonetheless, genetic factors, especially mutations in genes like the Ret proto-oncogene (RET) and endothelin receptor type B (EDNRB), add complexity to HSCR's origins [3]. Additionally, HSCR patients are susceptible to Hirschsprung-associated enterocolitis (HAEC) [4–6]. Moreover, recent epidemiological studies have reported an increased incidence of inflammatory bowel disease (IBD) among HSCR patients [4, 6–8]. These recent studies raise questions such as: Could there be a shared pathophysiology between HSCR, HAEC, and IBD? Do the similarities in apoptotic and necroptotic pathways point to common underlying mechanisms? Are the treatment responses in HSCR and CO cells indicative of broader disease progression connections?

At the molecular level, the receptor interacting protein kinase (RIPK) family, particularly RIPK1 and RIPK3, appears to be a promising target. These kinases are crucial for maintaining intestinal barrier integrity and regulating cell death mechanisms. The signaling pathways mediated by RIPKs, which shift from cell survival to apoptotic (RIPK1-dependent) or necroptotic cell death (RIPK3-dependent), may present a connection since dysregulation of these pathways has been documented in IBD [9].

Using flow cytometry and directly conjugated monoclonal antibodies, we analyzed cell death pathways in patient-derived organoid cultures from HSCR patients and controls. To achieve this, we refined an assay to measure various pathways using FACS in organoid cultures [10].

This method enabled us to differentiate between cell death mechanisms within cell populations derived from colonic organoids. Consequently, we identified previously unnoticed, yet significant small and large cell groups undergoing various stages of RIPK1-dependent apoptosis (RIPK1 +), RIPK1-independent apoptosis (Caspase-3 +), and necroptosis (RIPK3 +), in both viable and non-viable cells. Determining whether cells are viable (Zombie −) or non-viable (Zombie +) and their respective cell death pathways is critical for diagnosing diseases and understanding their progression. For example, in gastrointestinal diseases, the balance between viable and non-viable epithelial cells significantly affects the intestinal barrier’s integrity [11]. In this study, we observed that a proinflammatory cocktail containing TNF-α, IL-6, and IL-1β can simultaneously induce multiple forms of cell death within a single cell [12]. We demonstrated activated pathways exhibiting high levels of RIPK1-dependent apoptosis, RIPK1-independent apoptosis, and necroptosis [13].

## Materials and methods

### Establishment and maintenance of organoids

The study adhered to the Declaration of Helsinki guidelines and received approval from the Institutional Review Board Hamburg ethics committee (PV5251). Tissues from patients with Hirschsprung's disease (HSCR, *n* = 5) were sourced from surgically removed specimens, exclusively using the proximal ganglionic portion. Control tissues (CO, *n* = 5) were obtained from patients undergoing surgeries unrelated to Hirschsprung’s disease, ensuring a balanced comparison (Table 1). The samples were immediately placed in Iscove's modified Dulbecco's medium (IMDM, #12440053, Gibco Thermo Fisher Scientific, Waltham, MA, USA), supplemented with 20% fetal bovine serum (FBS, #0500-064, Thermo Fisher Scientific, Waltham, MA, USA) and 1% penicillin/streptomycin (P/S, #PS/B, Capricorn Scientific, Ebsdorfergrund, Germany). Afterward, they were thoroughly washed with sterile Dulbecco’s phosphate-buffered saline (DPBS; #37350, Gibco Thermo Fisher Scientific, USA), and the mucosal layer was carefully separated from underlying tissues, then cut into 1–2 mm pieces. Mucosal cells were isolated by incubating tissue slices in IMDM with 5 mM ethylenediaminetetraacetic acid (#15575–038, Invitrogen, Waltham, MA, USA) and 2 mM DL-dithiothreitol (DTT, #D9779-5G, Sigma-Aldrich, St. Louis, MO, USA) for 20 min at 4 °C, facilitating crypt structure isolation. They were further filtered through a 70 µm cell strainer (#352350, Corning, Corning, NY, USA) and washed in IMDM containing 2% FBS and 1% P/S, as well as Advanced Dulbecco’s modified Eagle medium (Advanced DMEM, #12491-015, Gibco Thermo Fisher Scientific, USA), which includes 1% 4-(2-hydroxyethyl)-1-piperazineethanesulfonic acid (HEPES, #H3537-100ML, Sigma-Aldrich, St. Louis, MO, USA), 1% GlutaMAX (#35050-061, Gibco Thermo Fisher Scientific, Waltham, MA, USA), and 1% P/S, collectively referred to as ‘Adv. +  +  + ’. Each washing step was followed by centrifugation at 500 × *g* and 4 °C for 10 min. A solution consisting of extracellular matrix in a 1:2 ratio (Cultrex UltiMatrix, #BME001, R and D Systems, Minneapolis, MN, USA) was prepared. The suspension was pipetted as 30 µl droplets into the center of each well in a 24-well plate (#3022521, Sarstedt AG and for 30 min to solidify the Cultrex. Subsequently, each well received 500 µl of Organoid Growth Medium Human (OGM-h, #060610, Stemcell Technologies, Canada), enriched with 5 mM ROCK-Pathway Inhibitor (ROC, #72302, Stemcell Technologies, Vancouver, Canada), 1% penicillin/streptomycin (P/S), and 0.02% Primocin (#ant-pm-05, InvivoGen, San Diego, CA, USA), with medium changed every 2–3 days.

**Table 1:** Patient characterristics for each sample used in this study

Sample	Gender	Age at operation (months)	Trisomy status	Diagnoses	Median group age
#1	Male	3.6	Negative	Hirschsprung’s disease	14.4
#2	Male	3.8	Positive	Hirschsprung’s disease, atrial septum defect, persistent oval foramen	
#3	Female	16.9	Negative	Hirschsprung’s disease	
#4	Female	14.4	Negative	Hirschsprung’s disease	
#5	Female	14.5	Negative	Hirschsprung’s disease	
#6	Female	6.2	Negative	Anal atresia, persistent oval foramen, kidney dysplasia, clubfoot	7.3
#7	Female	9.3	Negative	anal atresia, atrial septum defect, kidney dysplasia, hemivertebrae, multiple ribs, sacral dysplasia, heel feet	
#8	Male	7.3	Negative	anal atresia, hypospadia sine hypospadia	
#9	Male	4.9	Negative	ileo-colic invagination	
#10	Male	12.8	Negative	anal atresia, esophageal atresia, ventricular septum defect, fused kidneys, sacral agenesia	

After 7–10 days, organoids achieved the necessary confluence for passage. For dissociation, the Cultrex dome was mechanically disrupted in Adv. +  +  + , and the cell suspension was centrifuged at 300 × *g* and 4 °C for 5 min. Organoids were further broken down by pipetting 30–50 times. Following centrifugation for cell isolation and supernatant removal, cells were resuspended in Adv. +  +  + and embedded once again using Cultrex following the previously described protocol. Organoids were maintained in OGM-h containing 1% P/S, with the medium replaced every 2–3 days. Daily evaluations were conducted to monitor structural development and ensure their readiness for experimental use.

### Preparation of organoids for flow cytometry

**Fig. 1:**
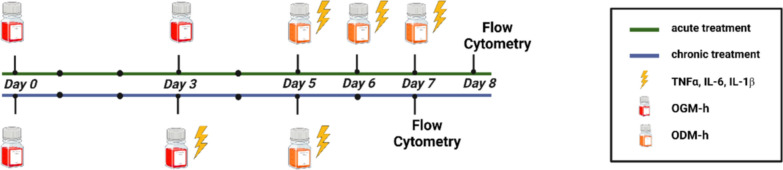
Experimental setup. Enteroids from both CO and HSCR were maintained in standard growth medium for 5 days and then differentiated until flow cytometry took place. Proinflammatory stimulation was achieved through a cytokine combination of TNFα, IL-1ꞵ, and IL-6 at days 3 and 5 for chronic and days 5, 6, and 7 for the acute regimen. Figure created with BioRender.com

Organoids were assigned to two different experimental conditions: acute and chronic treatment groups (Fig. 1). Within each group, samples were further divided into untreated and treated subsets. The treated subsets received a cytokine cocktail containing TNF-α (#300-01A, PeproTech, London, UK), IL-6 (#200-06, PeproTech, London, UK), and IL-1β (#200-01B, PeproTech, London, UK) at a concentration of 10 ng/ml for each cytokine, as determined by preliminary experiments [12]. All organoids were cultured in OGM-h until day 5. Thereafter, differentiation was induced by switching the medium to Organoid Differentiation Medium Human (ODM-h, #100-02114, Stemcell Technologies, Vancouver, Canada) with 1% P/S. In the chronic treatment protocol, treated organoids received the cytokine cocktail on days 3 and 5. In contrast, the acute treatment protocol involved administering the cytokine cocktail on days 5, 6, and 7. Untreated subgroups under both chronic and acute conditions received no cytokine cocktail.

For flow cytometry analysis, organoids were detached from Cultrex domes and resuspended in Adv. +  +  + . After centrifugation at 300 × g and 4 °C for 5 min, organoids were broken down into single cells using TrypLE (#12,605,028, Gibco Thermo Fisher Scientific, Waltham, MA, USA) at 37 °C for 4 min. Following incubation, cells were washed with Adv. +  +  + and centrifuged again at 300 × g and 4 °C for 5 min. The supernatant was discarded, and cell pellets were stained with Zombie NIR fluorescent dye (#423105, BioLegend, San Diego, CA, USA, dilution 1:2000) in DPBS for 30 min at 4 °C in the dark. Cells were then washed in DPBS and fixed using eBioFix intracellular fixation buffer (#00-8222-49, BD Biosciences, San Jose, CA, USA) for 20 min at 4 °C. Subsequently, cells were permeabilized with 0.25% Triton X-100 (#T8787, Sigma-Aldrich, St. Louis, MO, USA) for 15 min at 4 °C in darkness. Finally, cells were labeled with anti-RIPK3-Alexa Fluor 488 (clone B-2, #sc-374639AF488, Santa Cruz Biotechnology, Dallas, TX, USA, dilution 1:125) and anti-active caspase-3–BV650 (clone C92-605, #564096, BD Biosciences, San Jose, CA, USA, dilution 1:50) for 20 min at 4 °C in the dark.

### RIPK3–caspase-3 analysis using flow cytometry

Flow cytometric analysis was performed on 10,000 individual cells from each subgroup using the BD LSRFortessaTM (BD Biosciences, San Jose, CA, USA). Zombie NIR dye detection was facilitated by a 633 nm laser, with signal collection at 780/60 nm. Caspase-3–BV650 fluorescence was identified using a 405 nm laser and collected at 675/30 nm. RIPK3–Alexa Fluor 488 fluorescence was captured by a 488 nm laser and collected at 585/40 nm. Cell debris was excluded by gating based on forward scatter-area (FSC-A) versus side scatter-area (SSC-A) dot plots. Cells were further analyzed on a Zombie NIR versus SSC-A dot plot; all Zombie NIR-positive cells were considered dead. In contrast, Zombie NIR-negative cells, assumed to be viable, were gated on an FSC-A versus SSC-A plot. A more precise distinction between living and dead cells was achieved using caspase-3–BV650 versus RIPK3–Alexa Fluor 488 dot plots. Cells expressing caspase-3 alone were categorized as undergoing classic (RIPK1-independent) apoptosis, while those positive for only RIPK3 were classified as undergoing necroptosis. Cells positive for both caspase-3 and RIPK3 were interpreted as experiencing RIPK1-dependent apoptosis. Zombie + cells negative for both markers were presumed to follow an alternative cell death pathway. Fluorescence minus one (FMO) control was utilized for cell identification, gating, and color compensation. Data were analyzed using FlowLogic software version 8.7 (Inivai Technologies, Victoria, Australia).

### Light microscopy

Organoids were imaged using a Leica DM IL LED microscope at 4 × magnification. Images were taken directly in their culture wells without fixation or staining to preserve the native structure. Organoids were photographed before and after proinflammatory stimulation at specified time points to observe morphological changes, including crypt structure integrity and signs of cell death. Image analysis was conducted using ImageJ to evaluate structural alterations and overall morphology of the organoids.

### Immunofluorescence staining and confocal microscopy

Organoids were fixed in 4% paraformaldehyde, permeabilized with 0.5% Triton X-100, and blocked with 5% BSA to reduce non-specific antibody binding. They were then incubated with primary antibodies against β-catenin (1:200) and ZO-1 (1:200) overnight at 4 °C, followed by incubation with Alexa Fluor-conjugated secondary antibodies (1:500) for 1 h. DAPI (1 µg/mL) was used to stain nuclei. Confocal images were acquired using an Olympus FV 3000 for acute treatment and a Leica SP5 for chronic treatment, at 20 × magnification. Z-stack images provided detailed views of the organoid structure, highlighting the localization of β-catenin (red) and ZO-1 (green), with DAPI marking nuclei (blue). Image analysis was performed using ImageJ to assess changes in cellular morphology and junction integrity.

### Statistical analysis

Statistical analyses were conducted with GraphPad Prism version 10.1 (GraphPad Software, San Diego, CA, USA). Subgroup analysis employed the paired t test, while mean values and standard deviations (SD) were computed for each dataset. A *p* value less than 0.05 was considered to indicate statistical significance.

## Results

### Acute proinflammatory treatment causes increased cell death

**Fig. 2:**
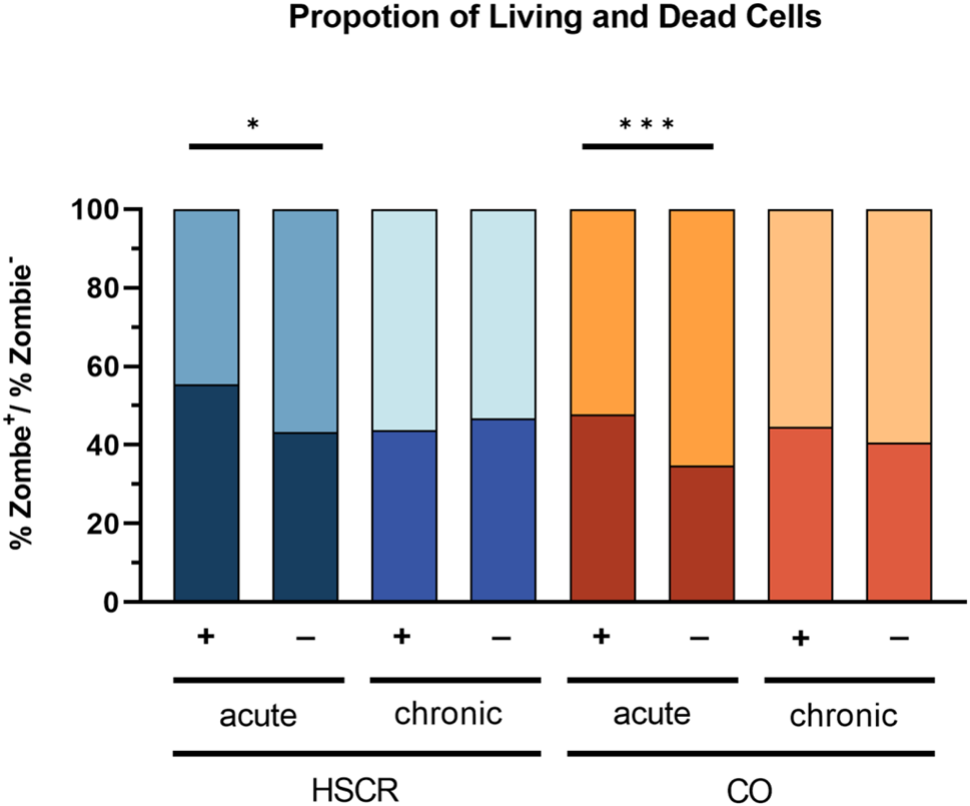
Proportions of dead and living cells. Analysis includes five enteroids for each measured subgroup. Cell proportions are shown for acute and chronic stimulation scheme before (−) and after treatment ( +). Whole cell population was separated according to their zombie status. Zombie^+^ cells were marked as dead. Zombie − cells were marked as alive and undergoing cell death. Analysis reveals a significant increase in dead cells after acute proinflammatory stimulation for both HSCR (*p* = 0.0029) and CO (*p* = 0.0009)

The analysis distinguished between non-viable and viable cells by their Zombie-status. Figure 2 shows a significant difference in the proportion of Zombie-positive cells between treated and untreated subgroups during acute stress. In the HSCR acute group, Zombie + cells were significantly increased in the treated subgroup (55.39 vs. 43.33%, *p* = 0.0229). Similarly, in the control acute group, treated subgroups had more Zombie + cells than untreated ones (47.71 vs. 34.70%, *p* = 0.009). However, under chronic stress, both HSCR and control groups showed a considerable number of dead cells before and after treatment, with no significant differences (Table 2).

**Table 2:** Summary of key findings on the effect of treatment on Zombie + (dead) cells in the acute HSCR and CO groups

Group		Zombie stain	Analysis group	Measurement	Key findings	*p* value
HSCR		Dead (Zombie^+^)	Acute	% dead cells	↑After treatment	0.0029
CO		Dead (Zombie^+^)	Acute	% dead cells	↑After treatment	0.009

### Death cells undergo RIPK1-independent apoptosis after proinflammatory stimulation

**Fig. 3:**
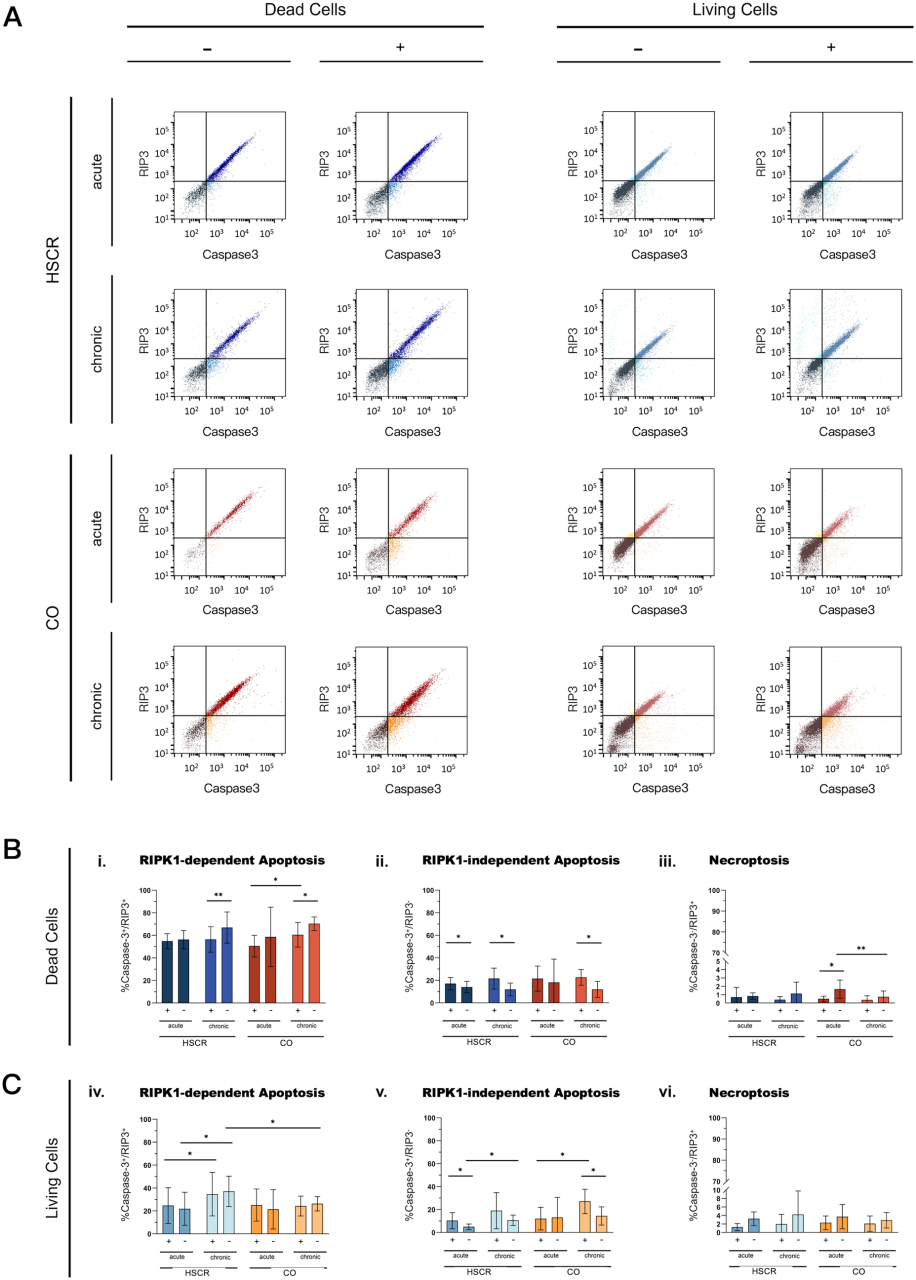
Summary of FACS results. A RIP3 versus caspase-3 dot plots for two exemplary samples from HSCR and CO with gating of RIP3-positive (upper left), caspase-3 positive (lower right), double-positive (upper right) and double-negative (lower left) cells. Analysis was performed for dead (left column) and living (right column) cell populations. Dot plots depict the changes in subgroup populations after treatment with either acute (upper row) or chronic (lower row) cytokine cocktail. B Dead cells show a decrease in RIPK1-dependent apoptosis after acute and chronic stimulation (**i**), while RIPK1-independent apoptosis increases after all treatment regimens (**ii**). Necroptosis was barely detectable and only showed significant changes for the CO groups (**iii**). C Living cells presented no significant changes for RIPK1-dependent apoptosis within the subgroups, but HSCR overall showed more living cells following chronic compared to acute stimulated subgroups (**iv**). Again, RIPK1-independent apoptosis increased after stimulation for most subgroups (**v**). Necroptosis played only a minor role in living cells undergoing cell death (**vi**)

RIPK1-dependent apoptosis (RIPK3 + /caspase-3 +): was observed as the main cell death mechanism in non-viable cells, as depicted in Fig. 3i. After acute treatment, HSCR organoids exhibited similar levels of double-positive cells (54.88 vs. 56.30%, *p* = 0.2825), while chronic stimulation resulted in a notable decrease in double positivity (66.95 vs. 56.42%, *p* = 0.0026). Similarly, CO organoids under chronic treatment showed a significant drop in double-positive cells (70.35 vs. 60.55%, *p* = 0.0407); however, acute treatment did not demonstrate significant changes, with RIPK3 + /caspase-3 + cells decreasing from 58.59 to 50.50% (*p* = 0.2094).

RIPK1-independent apoptosis (RIPK3^−^/caspase-3^+^). HSCR samples showed a significant increase in RIPK1-independent apoptosis after stimulation for both acute and chronic treatment (14.09 vs. 17.11%, *p* = 0.0220; 12.01 vs. 21.61%, *p* = 0.0158, respectively). While CO cells also presented with this pattern for chronic treatment, increasing from 11.93 to 22.59% (*p* = 0.0159), acute stimulation showed no affection (18.24 vs. 21.53%, *p* = 0.3140). HSCR and CO showed similar rates in caspase-3^+^ cells before and after treatment, as summarized in Fig. 3ii.

Necroptosis (RIPK3^+^/caspase-3^−^). Overall, necroptosis was just slightly detectable and showed no significance in HSCR cell death in both treatment regimens (Fig. 3iii). While chronic CO samples appeared similar, acute treatment of controls demonstrated a decline in necroptotic cells post-treatment from 1.66 to 0.50% (*p* = 0.0182).

The significant results of all the dead cell analysis are summarized in Table 3.

**Table 3:** Effects of treatment on the apoptotic and necroptotic pathways in Zombie + (dead) cells across chronic and acute HSCR and CO groups

Zombie status	Analysis group	Group	Measurement	Key findings	*p* value
Dead (Zombie^+^)	Chronic	HSCR	RIPK1-apoptosis (RIP3^+^/caspase-3^+^)	↓After treatment	0.0026
Dead (Zombie^+^)	Chronic	CO	RIPK1-apoptosis (RIP3^+^/caspase-3^+^)	↓After treatment	0.0407
Dead (Zombie^+^)	Acute	HSCR	Apoptosis (RIP3^−^/caspase-3^+^)	↑After treatment	0.0220
Dead (Zombie^+^)	Chronic	HSCR	Apoptosis (RIP3^−^/caspase-3^+^)	↑After treatment	0.0158
Dead (Zombie^+^)	Chronic	CO	Apoptosis (RIP3^−^/caspase-3^+^)	↑After treatment	0.0159
Dead (Zombie^+^)	Acute	CO	Necroptosis (RIP3^+^/caspase-3^−^)	↓After treatment	0.0182

Analysis reveals that RIPK1-dependent apoptosis is significantly reduced after chronic treatment, while increasing RIPK1-independent apoptotic processes at the same time. Still, RIPK1-dependent apoptosis was more prominent in chronic than in acute treated CO, but not in HSCR. RIPK1-independent apoptosis also increased after acute treatment in HSCR, but not in CO.

### Living cells undergo RIPK1-dependent apoptosis after proinflammatory stimulation

RIPK1-dependent apoptosis (RIPK3^+^/caspase-3^+^). RIPK1-dependent apoptosis remained the leading cause of cell death in still viable cells, but subgroup analysis was not able to detect significant changes for pre- and posttreatment, as summarized in Fig. 3iv. Following acute treatment, HSCR organoids showed slight decline after treatment (24.69 vs. 21.89%, *p* = 0.4065), and chronic stimulation led to a slight rise (34.67 vs. 37.12%, *p* = 0.7339). This was also observed for acute and chronic treated CO organoids (25.14 vs. 21.46%, *p* = 0.6885; 24.29 vs. 26.25%, *p* = 0.5594).

**RIPK1-independent apoptosis (RIPK3**^**−**^**/caspase-3**^**+**^**)**. As shown in Fig. 3v, there was a marked increase in cells undergoing apoptosis in the HSCR acute treated group, ranging from 4.93 to 10.35% (*p* = 0.0474), a trend mirrored in the HSCR chronic group though not reaching statistical significance (10.6 vs. 19.07%, *p* = 0.1195). For CO, the increase in caspase-3^+^ cells from 14.4 to 27.08% was significant (*p* = 0.0101) in the chronic condition. However, such a difference was not evident in the acute group. Overall, HSCR and CO showed similar amounts of caspase-3^+^ cells before and after stimulation.

**Necroptosis (RIPK3**^**+**^**/caspase-3**^**−**^**)**. The analysis revealed no significant differences in the detection of necroptotic cells neither between the HSCR and CO groups nor within their respective subgroups, as seen in Fig. 3vi. All significant results of this section are summarized in Table 4

**Table 4:** Impact of treatment on apoptosis in Zombie − (alive) cells in acute HSCR and chronic CO groups

Zombie status	Analysis group	Group	Measurement	Key findings	*P* value
Alive (Zombie^−^)	Acute	HSCR	Apoptosis (RIP3^−^/caspase-3^+^)	↑After treatment	0.0474
Alive (Zombie^−^)	Chronic	CO	Apoptosis (RIP3^−^/caspase-3^+^)	↑After treatment	0.0101

Treatment resulted an in increase in RIPK1-independent apoptosis after acute stimulation in HSCR and chronic stimulation in CO, but not vice versa.

### Light microscopy of intestinal organoids

**Fig. 4:**
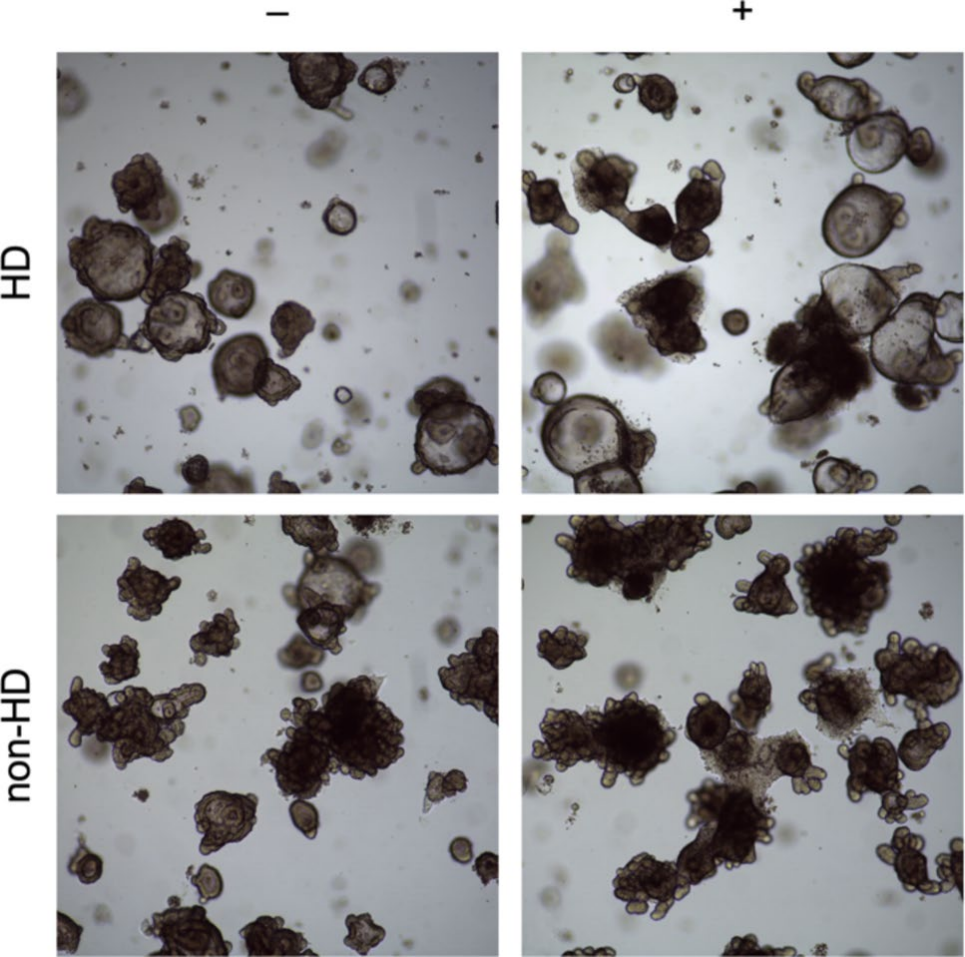
Light microscopy images of intestinal organoids from two patients, one suffering from Hirschsprung’s disease (HSCR) and one serving as non-HSCR control, before and after acute proinflammatory treatment. Tissue specimens prepared from colon sections, ganglionic in case of HSCR. Mucosa preparation, organoid isolation, and culture treatment were performed as described. Magnification 4×. Images taken with a Leica DM IL LED microscope. Acute proinflammatory stimulation results in increased cell death with disintegration of organoid structure and signs of cell death. Complexity of overall organoids morphology as seen before stimulation is mainly maintained

Under light microscopy, organoids from both HSCR patients and non-HSCR controls initially presented with well-defined crypt structures and complex morphology. After acute proinflammatory stimulation with TNF-α, IL-6, and IL-1β, HSCR organoids displayed a marked increase in cell death, characterized by visible disintegration and loss of structural integrity. Chronic stimulation led to further changes in the studied organoids, resulting in a rounded shape with thickened walls, suggesting a progressive loss of crypt structure complexity (Fig. 4**).**

### Immunofluorescence staining of organoids and confocal microscopy analysis

**Fig. 5:**
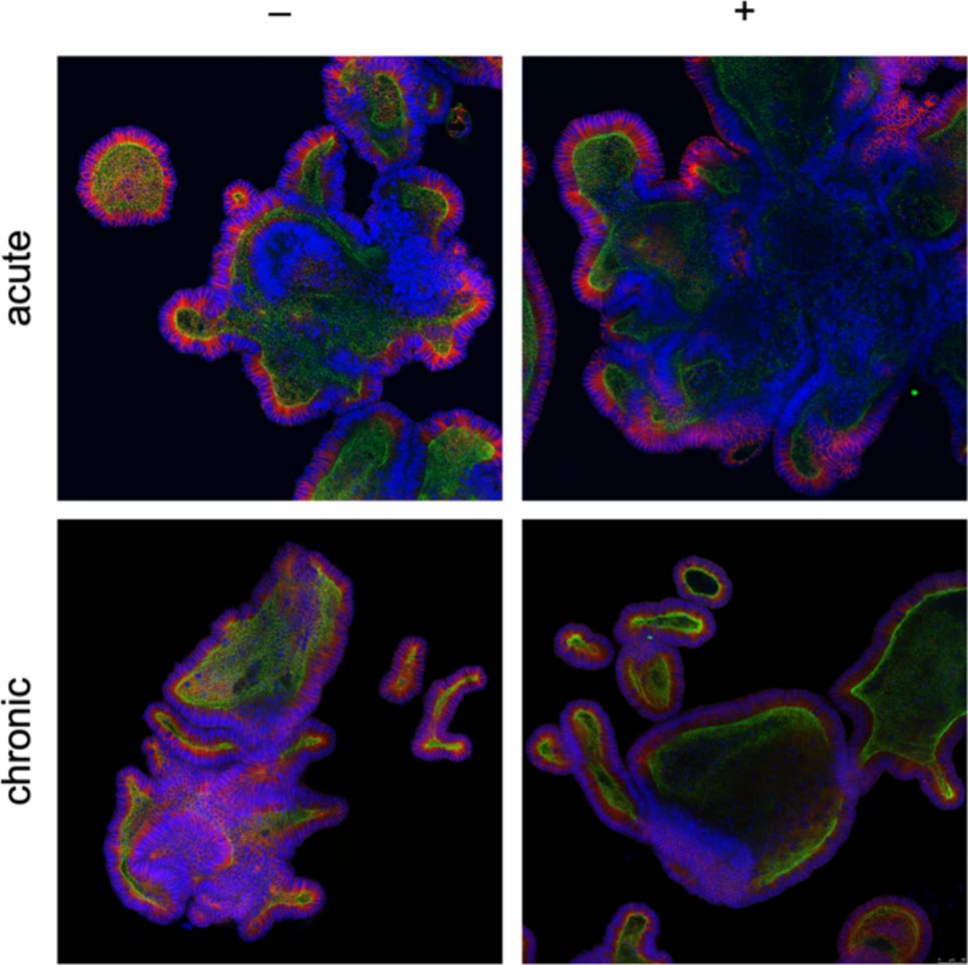
Immunofluorescence microscopy of intestinal organoids from a patient with Morbus Hirschsprung. Tissue specimens prepared from ganglionic colon section. Mucosa preparation, organoid isolation, and culture treatment were performed as described. Outlining of the organoids marked by intestinal barrier markers beta-catenin (red) and ZO-1 (green), nuclei stained with DAPI (blue) for orientation. Magnification 20×, images taken with the Olympus FV 3000 (acute) and Leica SP5 (chronic) confocal microscopes. Before stimulation, enteroids present with defined crypt structures and complex morphology. Acute treatment with proinflammatory cytokines leads to increased cell death, seen by nuclei destruction and loss of outlines. Following chronic treatment, organoids lost most of the crypt structure complexity and presented round with thickened wall diameter

Immunofluorescence staining with β-catenin and ZO-1 revealed a strong respond toward proinflammatory inflammation. Before stimulation, the studied organoids exhibited strong expression of β-catenin (red) and ZO-1 (green), indicating intact cell junctions and well-organized epithelial layers. Following acute proinflammatory treatment, organoids showed disrupted β-catenin and ZO-1 staining, with a diffuse and disorganized pattern suggestive of compromised cell junctions and increased permeability. DAPI staining revealed fragmented nuclei, indicating apoptotic cell death. Confocal microscopy provided a detailed assessment of the alterations in organoid structure following proinflammatory stimulation. Acute treatment in HSCR organoids led to notable nuclear destruction, as evidenced by DAPI staining, and a loss of β-catenin and ZO-1 outlines, indicating compromised barrier function. Chronic stimulation exacerbated these effects. Z-stack imaging showed that in chronic conditions, the HSCR organoids lost much of their crypt architecture and appeared to have increased wall thickness, suggesting a reactive remodeling in response to sustained inflammatory stress (Fig. 5).

## Discussion

The introduction of this assay into spheroid cultures marks a significant advancement in the study of cell death, providing a nuanced understanding of apoptosis and revealing the role of RIPK1 in non-viable cells. By enabling a detailed examination of cell death phenotypes in both viable and non-viable populations, this technique allows for the identification of classic apoptosis through caspase-3 positivity and the elucidation of RIPK1-mediated mechanisms [12]. Utilizing a proinflammatory cocktail of TNF-α, IL-6, and IL-1β to mimic the inflammatory environment of inflammatory bowel disease (IBD), we observed clear differences in the cellular responses to acute versus chronic inflammation [11]. Light microscopy (Fig. 4**)** and immunofluorescence (Fig. 5) of intestinal organoids derived from patients with Hirschsprung's disease (HSCR) demonstrated profound structural alterations under proinflammatory conditions. Initially, the organoids exhibited well-defined crypt structures and complex morphology. However, acute proinflammatory stimulation led to a marked increase in cell death, indicated by nuclear destruction and a loss of cellular outlines. Chronic stimulation exacerbated this effect, resulting in a significant reduction in structural complexity, with organoids becoming rounded and developing thicker walls. HSCR organoids displayed considerable disintegration and cell death. These observations indicate that HSCR organoids have a unique and compromised response to inflammatory stress, highlighting an altered epithelial barrier and immune function in the disease. This work not only underscores the vulnerability of HSCR tissues to inflammatory stimuli, but also emphasizes the critical role of inflammatory pathways in the pathophysiology of HSCR.

Under acute conditions, HSCR specimens exhibited an increased number of dead cells (Zombie +) compared to chronic stimulation, a pattern also seen in control specimens. This suggests an adaptive cellular response over time, transitioning toward survival mechanisms. Additionally, although not statistically significant, HSCR tissues had more dead cells than controls, indicating distinct responses to inflammatory challenges in HSCR.

Distinguishing between RIPK1-independent and RIPK1-dependent apoptosis is crucial due to their differing biological implications [13]. RIPK1-independent apoptosis is typically immunologically silent, avoiding an inflammatory response. In contrast, RIPK1 functions as both a kinase and scaffold, balancing cellular survival, apoptosis, and necroptosis [9, 10]. Its activation can shift cellular fate toward inflammatory pathways, especially when influenced by proinflammatory cytokines such as TNF-α, IL-6, and IL-1β [14]. Clinically, aberrant RIPK1 signaling is associated with inflammatory conditions such as IBD, where it regulates the transition of NF-κB signaling from cell survival to apoptosis or necroptosis [9, 10]. This dysregulation disrupts the intestinal barrier, allowing bacterial invasion and leading to enterocolitis. Given the histological similarity between IBD (particularly ulcerative colitis) and HAEC, it is plausible that aberrant RIPK1 signaling also contributes to HAEC pathogenesis [9, 15, 16].

In the context of HSCR and HAEC, altered apoptotic pathways may explain the differential responses to inflammatory stimuli. Our study shows that in HSCR, cells are more inclined towardsRIPK1-dependent apoptosis, which can link to proinflammatory signaling. This contrasts with the cellular environment in healthy controls, where apoptosis occurs in a more RIPK1-independent manner, often avoiding inflammatory escalation [17]. Furthermore, serum-derived exosomal microRNA-18a-5p has been shown to promote apoptosis in HAEC through the suppression of RORA and regulation of the SIRT1/NFκB pathway, creating a proinflammatory microenvironment [18]. Additionally, disruptions in neuroimmune regulation have been observed, suggesting a complex interplay between nerve cells and immune cells in HAEC, which could further drive the unique apoptotic landscape in these patients [19]. This evidence underscores that in HSCR, apoptosis and its regulation via RIPK1 may play a more prominent role in the pathogenesis of HAEC, diverging significantly from the mechanisms observed in healthy controls.

Understanding the dynamics between viable and non-viable cells is crucial, especially in how a proinflammatory stimulus can activate different cell death pathways within the same cell. A viable cell, despite initiating cell death processes, may initially balance these mechanisms depending on factors like RIPK1 availability. Over time, this balance shifts toward non-viability, progressing through apoptosis or necroptosis. Our analysis showed that viable cells in the HSCR group more frequently underwent RIPK1-dependent apoptosis, especially under chronic stress, whereas control group cells showed no difference between acute and chronic conditions. This suggests a cellular shift in HSCR toward classic apoptosis when RIPK1 becomes less available over time upon proinflammatory stimulation (Table 3**, **Fig. 3v). In non-viable cells, a more pronounced regulatory role for RIPK1 was observed, as evidenced by a decrease in RIPK1-dependent apoptosis upon chronic stimulation. This adaptive modulation in apoptotic pathways indicates that cells, under prolonged stress, may recalibrate their mechanisms to avoid the inflammatory pathways initially triggered by RIPK1 [20]. This shift could represent an attempt by the tissue to adapt to sustained damage or a progression toward a more critical pathological state [21].

The low presence of necroptotic cells (RIPK3 + /caspase-3−) across both HSCR and control groups further emphasizes that necroptosis, a highly proinflammatory cell death process, plays a subordinate role in this experimental context (Fig. 3C vi). Necroptosis, characterized by cell membrane rupture and subsequent tissue inflammation, is evidently not a major driver of the observed pathology in HSCR or its controls upon stimulation in this setting [13].

## Conclusion

In summary, our study highlights the complex interplay of apoptotic pathways in response to inflammatory stimuli in both acute and chronic settings. It emphasizes the importance of considering cell viability when examining cellular responses, particularly in pathological conditions like HSCR and HAEC, where altered apoptosis plays a key role. RIPK1 emerges as a pivotal regulatory protein, influencing the threshold between apoptosis and necroptosis and thereby affecting the overall balance of cell death. This nuanced understanding of cellular adaptation to proinflammatory environments underscores the multifaceted nature of inflammatory responses and opens avenues for further research into the modulation of these pathways for therapeutic benefit.

## Abbreviations

CO: Control group
FSC-A: Forward scatter area
HAEC: Hirschsprung-associated enterocolitis
HSCR: Hirschsprung's disease
IBD: Inflammatory bowel disease
IL-1β: Interleukin 1 beta
IL-6: Interleukin 6
ODM-h: Organoid differentiation medium human
OGM-h: Organoid growth medium human
RIPK1: Receptor-interacting protein kinase 1
RIPK3: Receptor-interacting protein kinase 3
SSC-A: Side scatter area
TNFR1: Tumor necrosis factor receptor 1
TNF-α: Tumor necrosis factor alpha

## Declarations

### Content of publisher

All claims expressed in this article are solely those of the authors and do not necessarily represent those of their affiliated organizations, or those of the publisher, the editors, and the reviewers. Any product that may be evaluated in this article, or claim that may be made by its manufacturer, is not guaranteed or endorsed by the publisher.

### Data availability

The original contributions presented in the study are included in the article/Supplementary Material. Further inquiries can be directed to Christian Tomuschat.
